# Assessing the accuracy of multiparametric MRI to predict clinically significant prostate cancer in biopsy naïve men across racial/ethnic groups

**DOI:** 10.1186/s12894-022-01066-9

**Published:** 2022-07-18

**Authors:** Julio Meza, Rilwan Babajide, Ragheed Saoud, Jamila Sweis, Josephine Abelleira, Irene Helenowski, Borko Jovanovic, Scott Eggener, Frank H. Miller, Jeanne M. Horowitz, David D. Casalino, Adam B. Murphy

**Affiliations:** 1 Department of Urology, Northwestern University, 710 N. Fairbanks Court Olson Pavilion 8-250, Chicago, IL 60611 USA; 2 University of Chicago Division of Urology, Chicago, IL USA; 3 Northwestern University Department of Radiology, Chicago, IL USA; 4 Arthur Smith Institute of Urology at Riverhead, Northwell Health, Riverhead, NY USA

**Keywords:** Prostate cancer, PIRADS, MRI, Sensitivity, Race, Ethnicity, Comparative effectiveness

## Abstract

**Introduction:**

The Prostate Imaging Reporting and Data System (PIRADS) has shown promise in improving the detection of Gleason grade group (GG) 2–5 prostate cancer (PCa) and reducing the detection of indolent GG1 PCa. However, data on the performance of PIRADS in Black and Hispanic men is sparse. We evaluated the accuracy of PIRADS scores in detecting GG2-5 PCa in White, Black, and Hispanic men.

**Methods:**

We performed a multicenter retrospective review of biopsy-naïve Black (n = 108), White (n = 108), and Hispanic (n = 64) men who underwent prostate biopsy (PB) following multiparametric MRI. Sensitivity and specificity of PIRADS for GG2-5 PCa were calculated. Race-stratified binary logistic regression models for GG2-5 PCa using standard clinical variables and PIRADS were used to calculate area under the receiver operating characteristics curves (AUC).

**Results:**

Rates of GG2-5 PCa were statistically similar between Blacks, Whites, and Hispanics (52.8% vs 42.6% vs 37.5% respectively, *p* = 0.12). Sensitivity was lower in Hispanic men compared to White men (87.5% vs 97.8% respectively, *p* = 0.01). Specificity was similar in Black versus White men (21.6% vs 27.4%, *p* = 0.32) and White versus Hispanic men (27.4% vs 17.5%, *p* = 0.14).

The AUCs of the PIRADS added to standard clinical data (age, PSA and suspicious prostate exam) were similar when comparing Black versus White men (0.75 vs 0.73, *p* = 0.79) and White versus Hispanic men (0.73 vs 0.59, *p* = 0.11). The AUCs for the Base model and PIRADS model alone were statistically similar when comparing Black versus White men and White versus Hispanic men.

**Conclusions:**

The accuracy of the PIRADS and clinical data for detecting GG2-5 PCa seems statistically similar across race. However, there is concern that PIRADS 2.0 has lower sensitivity in Hispanic men compared to White men. Prospective validation studies are needed.

**Supplementary Information:**

The online version contains supplementary material available at 10.1186/s12894-022-01066-9.

## Introduction

Prostate cancer (PCa) is the most commonly diagnosed malignancy in men [1]. Early detection of PCa is important, as it has been shown to reduce cancer-specific mortality [2]. However, this benefit comes at the cost of over detection and overtreatment of indolent Gleason grade group 1 (GG1) PCa. The classic diagnostic pathway for PCa involves a digital rectal exam (DRE), PSA testing, and transrectal ultrasound guided (TRUS) prostate biopsy (PB). However, both DRE and PSA lack specificity for clinically significant GG2-5 PCa, and TRUS-PB suffers from low sensitivity for PCa due to its limited ability to sample the anterior region of the prostate as well as overdiagnosis of GG1 PCa [3, 4]. Given the limitations of TRUS-PB, the risks associated with it, including bleeding and sepsis, and the healthcare costs it generates, there is a need for a non-invasive screening test for aggressive PCa in order to limit the number of unnecessary biopsies performed [5, 6].

Accordingly, multiparametric magnetic resonance imaging of the prostate (mpMRI) has emerged as an important tool to enhance the detection of clinically significant Gleason grade group (GG) 2–5 PCa and guide targeted prostate biopsies while reducing the detection of GG1 PCa [7, 8]. Clinically, the Prostate Imaging Reporting and Data System (PIRADS) score from the MRI is being used as a binary test; patients with a PIRADS 3–5 lesion will most likely have a systematic and targeted PB while a patient with a PIRADS 1 or 2 lesion will have the biopsy deferred. Since PIRADS is used a binary test, a high sensitivity becomes increasingly critical to avoid false negatives. Even though there are various studies that have validated the high sensitivity of mpMRI, these studies are predominately compromised of White participants and there has been little to no validation of PIRADS in Hispanic men [9, 10]. We sought to compare the overall accuracy of mpMRI by comparing the area under the receiver operating characteristics curve (AUC) and to compare the sensitivity of the clinically utilized PIRADS ≥ 3 threshold between biopsy-naïve Black, White, and Hispanic men.

## Methods

We performed a multicenter retrospective review of 280 biopsy-naïve patients who underwent PB following mpMRI between May 2014 and January 2021 at a single academic medical center or between October 2018 and February 2021 at a second academic medical center. Patients were gathered using the Enterprise Data Warehouse at the first academic medical center for men diagnosed with elevated PSA and who underwent an MRI of the prostate. Men at the second academic medical center were gathered from an IRB-approved prospectively collected biopsy database from one physician. All men were referred to the outpatient Urology clinics at these institutions for elevated PSA. A subset of men was found to have abnormal digital rectal exam (DRE) at the time of prostate biopsy and is included. We excluded men with prior negative biopsies using pathology reports and clinical urology progress notes. Following institutional review board approval (IRB ID: STU00211583), patient demographic and clinical data including age, race, PSA before PB, DRE results, prostate volume, PIRADS scores, and pathology results from PB were abstracted from the electronic medical record. PSA density was calculated as the quotient of serum PSA (ng/ml) and MRI prostate volume (cm^3^).

MpMRI examinations were performed using 3 T MR scanners (Siemens Medical System, Erlangen, Germany) with triplanar T2 weighted, axial dynamic contrast-enhanced (DCE), and axial diffusion-weighted imaging (DWI) using a phased-array body coil without endorectal coil. Parameters for T2 weighted imaging include slice thickness 3 mm, field of view 200 × 200 mm, matrix 307 × 384 or 288 × 320, and voxel size 0.65 × 0.52 mm or 0.69 × 0.62 mm. Parameters for DWI imaging include slice thickness was 4 mm, field of view 228 × 228 cm, matrix 114 × 114, and voxel size 2.0 × 2.0 mm. Diffusion-weighted sequences were acquired with b values of 50, 500, 1000, and 1600, and b value imaging and the ADC maps were analyzed qualitatively. Parameters for DCE imaging included slice thickness 4 mm, field of view 220 × 220 mm, matrix 128 × 128, and voxel size 1.72 × 1.72 mm. The temporal resolution for DCE was 10.5 s. Dynamic contrast enhanced imaging postprocessing was performed on DynaCAD Prostate (Philips, Massachusetts, United States). All mpMRI studies were interpreted by expert fellowship trained genitourinary radiologists. Ninety-two percent of MRIs were read using PIRADS version 2.0 and sensitivity between version 2.0 and 2.1 is nearly identical [11].

Eighty percent of biopsies were UroNav® MRI-US Fusion Transrectal Ultrasound (TRUS) biopsies with systematic and target cores taken of the prostate and this did not vary by race. Eighteen percent of biopsies were TRUS guided biopsies. Two percent of biopsies were Cognitive Fusion TRUS prostate biopsies.

Only patients who self-reported as non-Hispanic White, non-Hispanic Black, or Hispanic were included in the study. Patients with a history of previous PCa, prior PB, prostate volume greater than 100 cm^3^ on MRI, PSA greater than 15.0 ng/mL, or who underwent PB more than 18 months after mpMRI were excluded. During the initial EDW query, 1390 patients were gathered during the initial EDW query at the first institution and 11 patients were gathered from the second institution. Of those there were only 108 Black and 64 Hispanic patients eligible. We then matched 108 White patients by age within 3 years and PSA in ranges 0–5.0, 5.1–10.0, and 10.1–15.0 to our 108 Black patients by randomly selecting White patients from the first institution until we had 108 matched White patients.

Pathological assessment of biopsy specimens was performed by expert uro-pathologists in accordance with the 2014 International Society of Urological Pathology Consensus Conference [12]. Clinically significant prostate cancer was defined as GG2-5 PCa.

All results were reported by racial/ethnic groups as White, Black and Hispanic; Hispanic White and Hispanic Black participants are included among Hispanics which is consistent with the US Census, NIH, and other PIRADS studies reporting of race and ethnicity [13]. Methods for self-reporting were consistent throughout the entire study. The electronic medical record system had eight categories for race: American Indian or Alaska Native, Asian, Black or African American, Declined, Native Hawaiian or Other Pacific Islander, Other, Unable to Answer, and White. There were four categories for ethnicity: Hispanic or Latino, Not Hispanic or Latino, Declined, Unable to Answer. There was an option to choose up to three races, however, everyone in our cohort only chose one race. Continuous data were reported as medians with interquartile range (IQR). Independent-Samples Median tests were used to compare continuous variables by racial/ethnic group. Comparisons of the categorical variables were made using Chi-square tests. The proportion of GG2-5 PCa within PIRADS groups (PIRADS 1–2, 3, 4, 5) was stratified by race/ethnicity.

The accuracy of mpMRI for detecting GG2-5 PCa was measured by constructing the receiver operating characteristic (ROC) curves and comparing the area under the ROC curves (AUC) and their 95% confidence intervals. The models utilized included: 1) Clinical variables Model (Age, Log_2_[PSA], and suspicious DRE, 2) PIRADS alone 3) Clinical variables model + PIRADS. Kolmogorov Smirnov tests were used to compare non-parametric AUCs between racial groups as well as between models within racial groups. The PSA used in the models was the total serum PSA that triggered the biopsy or referral. DRE was coded as a binary variable (normal/non-palpable/not performed vs suspicious). PIRADS was coded as an ordinal 4-level variable with PIRADS = 1–2; = 3, = 4, and = 5. Specifically, we compared AUC(Clinical variables), AUC(PIRADS) and AUC (Clinical variables + PIRADS) across race between White men versus Black men and White men versus Hispanic men.

For assessment of the sensitivity and specificity for the detection of GG2-5 PCa, a PIRADS ≥ 3 was used as the threshold for a positive test. We had 80% Power to detect a 0.150 difference in AUC between White and Hispanic patients and a 0.134 difference in AUC between White and Black patients. Based on a pooled sensitivity of PIRADS version 2.0 and 2.1 for GG2-5 PCa of 97.0% we had 80% Power to detect a 11.2% difference in sensitivity between White and Hispanic patients and a 10.2% difference in sensitivity between White and Black patients [14, 15].

All comparisons were two‐sided and *p* values < 0.05 indicated statistical significance. Statistical analysis was performed using SPSS 28 (IBM Corporation, Armonk, NY) and R 4.0.3. All procedures were performed in accordance with the Declaration of Helsinki.

## Results

A total of 280 men were included in this study, of which 108 (38.6%) were Black, 108 (38.6%) were White, and 64 (22.8%) were Hispanic (see Table 1). There were significant differences between Black, White and Hispanic men in medians for BMI (29.3 vs 26.9 vs 27.3 kg/m^2^, *p* = 0.001), PSA (6.5 vs 5.3 vs 4.7 ng/mL, *p* < 0.001), PSA density (0.14 vs 0.13 vs 0.09 ng/mL/cm^3^, *p* = 0.006) and family history of PCa (16.7% vs 25% vs 9.4%, *p* = 0.03), respectively. Additionally, there were differences in the rates of GG1-5 PCa detection on PB (69.4% vs 64.8% vs 46.9%, *p* = 0.01). However, rates of GG2-5 PCa were statistically similar (52.8% vs 42.6% vs 37.5%, *p* = 0.12) between racial/ethnic groups. Furthermore, there were no differences in age, frequency of abnormal DRE, PIRADS scores, or time between mpMRI and PB between racial/ethnic groups. Additional clinical and demographic characteristics are reported in Table 1.

**Table 1:** Patient characteristics by race/ethnicity

Continuous variables	Black (n = 108)	White (n = 108)	Hispanic (n = 64)	*p* value^a^
	Median [IQR]			
Age, years	61.0 [55.2, 67.0]	64.0 [57.2, 69.0]	61.5 [57.2, 69.0]	0.160
BMI, kg/m^2^	29.3 [26.2, 32.3]	26.9 [24.4, 29.1]	27.3 [25.1, 31.2]	**0.001**
PSA, ng/mL	6.5 [4.8, 8.1]	5.3 [4.6, 7.0]	4.7 [4.1, 6.2]	** < .001**
MRI prostate volume, cm^3^	40.2 [30.3, 59.8]	43.6 [32.2, 60.4]	50.5 [34.0, 83.6]	0.222
PSA density, ng/mL/cm^3^	0.14 [0.09, 0.24]	0.13 [0.09, 0.19]	0.09 [0.06, 0.15]	**0.006**
Time between MRI and Biopsy, days	29.0 [19.2, 45.0]	26.5 [16.2, 45.5]	32.5 [17.2, 53.0]	0.231
MRI Lesions	1.0 [1.0, 2.0]	1.0 [1.0, 2.0]	1.0 [1.0, 2.0]	0.410
Systematic Cores	13.0 [10.0, 16.0]	13.0 [9.0, 16.0]	13.0 [10.0, 16.0]	0.587
Target Cores	3.5 [3.0, 6.0]	4.0 [3.0, 6.0]	3.0 [2.0, 4.75]	**0.016**
Systematic Cores with GG 2–5 PCa	0.0 [0.0, 2.0]	0.0 [0.0, 3.0]	0.0 [0.0, 1.75]	0.294
Target Cores with GG 2–5 PCa	0.0 [0.0,2.0]	0.0 [0.0, 2.0]	0.0 [0.0, 1.75]	**0.047**
Maximum GG 2–5 PCa Length in Systematic Cores, cm	0.27 [0.1, 0.6]	0.58 [0.2, 0.9]	0.5 [0.1, 1.0]	**0.02**
Maximum GG 2–5 PCa Length in Target Cores, cm	0.6 [0.3, 0.8]	0.7 [0.2, 1.0]	0.8 [0.2, 1.1]	0.353

Categorical variables	n (%)	n (%)	n (%)	*p* value^b^
Family History of PCa	18 (16.7%)	27 (25.0%)	6 (9.4%)	**0.03**
5-Alpha reductase	1 (0.9%)	5 (4.6%)	4 (6.3%)	0.14
Abnormal DRE	5 (4.6%)	14 (13.0%)	4 (6.3%)	0.67
Used PIRADS Version 1.0	4 (3.7%)	3 (2.8%)	0 (0.0%)	**< 0.001**
Used PIRADS Version 2.0	100 (92.6%)	105 (97.2%)	54 (84.4%)	
Used PIRADS Version 2.1	4 (3.7%)	0 (0.0%)	10 (15.6%)	
PIRADS 1–2	13 (12.0%)	18 (16.7%)	10 (15.6%)	0.61
PIRADS 3	21 (19.4%)	22 (20.4%)	22 (34.4%)	0.05
PIRADS 4–5	74 (68.5%)	68 (63.0%)	32 (50.0%)	0.05
GG 1–5 PCa	75 (69.4%)	70 (64.8%)	30 (46.9%)	**0.01**
GG 2–5 PCa	57 (52.8%)	46 (42.6%)	24 (37.5%)	0.12

^a^Using Independent-Samples Median Test

^b^Using χ^2^ tests. Age was at the time of index biopsy; BMI: Body Mass Index; DRE: Digital Rectal Exam; PCa: Prostate Cancer; PIRADS: Prostate Imaging Reporting and Data System; PSA: Prostate Specific Antigen. PSA was doubled if the patient was taking a 5-alpha reductase inhibitor for at least 3 months. GG: Gleason grade group. Cores are the prostate biopsy cores. All Continuous variables is data per prostate

A total of 3,540 systematic PB cores were taken, of which 1324 (37.4%) were from Black men, 1361 (38.4%) were from White men and 855 (24.2%) were from Hispanic men. The total number of target PB cores taken was 1,031, of which 390 (37.8%) were from Black men, 423 (41.0%) were from White men and 218 (21.1%) were from Hispanic men. The were no differences between race in medians for MRI lesions, systematic cores, systematic cores with GG 2–5 PCa and the maximum length of GG 2–5 PCa in target cores. However, there were significant differences between Black, White and Hispanic men in medians for target cores taken (3.5 vs 4.0 vs 3.0, *p* = 0.016), target cores with GG 2–5 PCa (0.0, IQR: 0.0–0.2 vs 0.0, IQR: 0.0–0.2 vs 0.0, IQR: 0.0–1.75, *p* = 0.047) and maximum length of GG 2–5 PCa in systematic cores (0.27 vs 0.58 vs 0.5, *p* = 0.02), respectively.

Table 2 illustrates the detection rate of GG2-5 PCa within PIRADS groups by racial/ethnic group. In Black, White, and Hispanic men, rates of GG2-5 PCa generally increased with increasing PIRADS scores. Comparing Black men versus White men and White men versus Hispanic men, there was not a statistically significant difference in incidence of GG2-5 PCa for PIRADS 1–2 (low suspicion) lesions (15.4% vs 5.6%, *p* = 0.56; 5.6% vs 30.0%, *p* = 0.12), PIRADS 3 (equivocal suspicion) lesions (28.6% vs 18.2%,*p* = 0.49; 18.2% vs 31.8%, *p* = 0.49), PIRADS 4 (high suspicion) lesions (64.8% vs 62.5%, *p* = 0.80; 62.5% vs 40.7%, *p* = 0.06), or PIRADS 5 (very high suspicion) lesions (70.0% vs 50.0%, *p* = 0.26; 50.0% vs 60.0%, *p* = 0.99).

**Table 2:** Rates of GG2-5 PCa within PIRADS groups by race. Black and Hispanic men compared to White men

PIRADS	White Proportion of men with GG2-5 PCa by PIRADS group (%)	Black Proportion of men with GG2-5 PCa by PIRADS group (%)	*p* value^a^	Hispanic Proportion of men with GG2-5 PCa by PIRADS group (%)	*p* value^a^
	n = 108	n = 108		n = 64	
1–2	1/18 (5.6%)	2/13 (15.4%)	0.56	3/10 (30.0%)	0.12
3	4/22 (18.2%)	6/21 (28.6%)	0.49	7/22 (31.8%)	0.49
4	35/56 (62.5%)	35/54 (64.8%)	0.80	11/27 (40.7%)	0.06
5	6/12 (50.0%)	14/20 (70.0%)	0.26	3/5 (60.0%)	1.00

*GG* Gleason grade group, *PIRADS* prostate imaging reporting and data system

^a^Comparison of the proportions in Black versus White and Hispanic versus White participants using χ^2^ tests

Table 3 shows the sensitivity and specificity for GG2-5 PCa, which were calculated for mpMRI by racial group using PIRADS ≥ 3 as the threshold for a positive test. Sensitivity was only significantly lower in Hispanic men when compared to White men (87.5% vs 97.8%, *p* = 0.01). Specificity was similar between Black and Hispanic men compared to White men.

**Table 3:** Sensitivity and specificity of PIRADS ≥ 3 for GG2-5 PCa by race

	Black (n = 108)	White (n = 108)	*p *value^a^	Hispanic (n = 64)	*p *value^a^
Sensitivity	96.5	97.8	0.57	87.5	**0.01**
Specificity	21.6	27.4	0.32	17.5	0.14

*GG* Gleason grade group, *PIRADS* prostate imaging reporting and data system

^a^*p* values are comparing proportions in Black and Hispanic men compared to White men. Using χ^2^ tests

ROC curves were created using three logistic regression models previously described, and AUCs were compared by racial group to assess the accuracy of the models in detecting GG2-5 PCa. There were no significant differences when comparing the AUCs of the clinical variables, PIRADS, and clinical variables + PIRADS models between Black and Hispanic men versus White men (Table 4). The model containing clinical variables + PIRADS performed moderately well in White men (0.73) and Black men (0.75) and relatively poorly in Hispanics (0.59); however, statistically there were no significant differences between White men and Hispanic men in GG2-5 PCa detection (0.73 vs 0.59, *p* = 0.11) or between White and Black men in GG2-5 PCa detection (0.73 vs 0.75, *p* = 0.79). De-identified patient data with corresponding legend is included in Additional file 1.

**Table 4:** Comparison of all models’ area under the curve by race/ethnic group

Model	Black AUC [95% CI]	White AUC [95% CI]	*p *value^a^	Hispanic AUC [95% CI]	*p *value^a^
Clinical variables	0.60 [0.50, 0.71]	0.55 [0.43, 0.66]	0.49	0.59 [0.45, 0.73]	0.64
PIRADS	0.70 [0.60, 0.80]	0.72 [0.63, 0.82]	0.76	0.58 [0.44, 0.73]	0.12
Clinical variables + PIRADS	0.75 [0.66, 0.84]	0.73 [0.64, 0.83]	0.79	0.59 [0.44, 0.74]	0.11

*AUC* area under the receiver operating characteristic curve, *PIRADS* prostate imaging reporting and data system

^a^*p* values correspond to comparison of AUCs across race/ethnic group using White patients as the referent group; *p* values were compared using Kolmogorov Smirnov Test; PIRADS was modeled as an ordinal variable with 4 values (i.e., 1–2, 3, 4, 5). The *p* values comparing the AUCs of the models within each race/ethnic group are all > 0.05

## Discussion

Multiparametric MRI (mpMRI) has become an important component in the diagnosis and surveillance of patients with PCa. The PIRADS scoring system allows for risk stratification of prostatic lesions and helps guide clinical decisions about the need for PB. However, it is used as a binary test to help patients avoid unnecessary PBs which relies upon a high sensitivity and specificity. Moreover, the accuracy of mpMRI to detect GG2-5 PCa in Hispanic and Black men is underreported within the literature. Our study evaluated and compared the sensitivity and specificity of mpMRI in non-Hispanic White, non-Hispanic Black, and Hispanic men. We demonstrate that the sensitivity of PIRADS in Hispanic men is significantly lower than the sensitivity of PIRADS in White men.

Black men in our cohort had a significantly higher serum PSA and PSA density than Whites and Hispanics, which is consistent with previously published data [16, 17]. However, AUC values of GG2-5 PCa, both overall and within PIRADS groups, were statistically similar between racial groups, though, in line with national trends where Blacks have higher incidence and Hispanics have lower incidence of PCa on biopsy relative to Whites.

One of the main reasons why urologists get secondary tests such as mpMRI is to avoid unnecessary PBs. The specificity of mpMRI to detect GG2-5 PCa has been reported around 56% in the general population, meaning that it helps about 1 out of 2 men avoid a PB [14]. In our Hispanic cohort, the specificity is only 17.5%. While not validated in Hispanics, blood-based biomarkers like density, PHI and 4Kscore have specificities that are higher than this, are less expensive, and take less time to perform. Avoiding unnecessary PB is critical due to the potential side effects of biopsy such as infection, bleeding, and erectile dysfunction [18]. While no head-to-head comparisons have been made in Hispanic populations, it warrants study. There is limited validation data in Hispanic participants, and we felt it important to provide the Hispanic data we had. Even though our Hispanic cohort only had 64 patients, we were still able to find a significantly lower sensitivity in Hispanic men compared to White men (87.5% vs 97.8%); implying that mpMRI misses 12.5% of GG2-5 PCa in Hispanic men.

Black men have been shown to have disproportionately worse prostate cancer specific health outcomes than White men, with a higher incidence (18.2% vs 13.3%) and mortality rate (4.4% vs 2.4%) [19–21]. While the specificity is only 21.6% in Black men, the sensitivity is greater than 95% and is likely worthwhile in identifying anterior tumors that maybe missed on systematic biopsy and are more common in Black men [22]. The benefits of MRI in Black men may outweigh the potentially lower specificity and avoidable biopsies.

Although the majority of patients had an mpMRI read using version 2.0 (92%), Hispanic patients had a higher proportion of mpMRIs read using version 2.1 and still had a significantly lower sensitivity. Our finding of a trend for lower sensitivity of PIRADS ≥ 3 largely using version 2.0 in Hispanic men is particularly important as a report by Hines et al. using PIRADS version 2.0 demonstrated that mpMRI has similar odds of detecting GG2-5 PCa in Hispanic men (OR 1.90, 95% CI 0.69–5.24, *p* = 0.22) compared to White men (OR 1.0) [13]. Larger sample sizes of Hispanic men would be needed to validate the lower sensitivity of mpMRI in Hispanic men.

The difference in sensitivity seen between Hispanic and White patients hinges on the high sensitivity we found in our cohort of White men (97.8%). However, the sensitivity found in the literature is also high. The sensitivity for PIRADSv2.0 ≥ 3 for GG2-5 PCa is 0.95 (95% CI 0.89–0.97) and for PIRADSv2.1 ≥ 3 for GG2-5 PCa is 0.94 (95% CI 0.88–0.97) [14, 23]. Therefore, the difference we found in sensitivity between Hispanic and White men has external validity. The cause of the lower sensitivity in Hispanic men is unknown.

On ROC curve analysis, the addition of PIRADS scores to the base model improved the accuracy of detection of GG2-5 PCa by 33% in Whites and 25% in Blacks, although it had no effect on AUC for Hispanics. Although adding PIRADS to the base model resulted in improvements in AUC for White and Black men, the AUCs for Black and Hispanic men using all models were statistically comparable to the AUC for White men. Nonetheless there may be a clinical difference in accuracy of PIRADS by racial group. Specifically, it appears to be less effective in detecting GG2-5 PCa in Hispanic men in this study. Given that this is the largest comparative effectiveness study for Hispanics, we felt it important to include them in this analysis. Our findings support those of Henning et al. [24] (n = 23 biopsy-naïve Blacks) and Walton et al. [25] (n = 5 biopsy-naïve Blacks) who found no difference in PCa detection using mpMRI in Black and White men in samples with varying mixtures of biopsy-naïve, prior negative biopsy and prior positive biopsy.

Although there were no differences in the number of MRI lesions across race, Hispanic men had the lowest number of target cores taken at a median of 3.0 per prostate. In comparison, White men had a median of 4.0 target cores taken per prostate. Three target cores taken per prostate is still an adequate amount of target cores taken since the median MRI lesions targeted is one for Hispanic men. Radical prostatectomy-based analysis has shown that prostate cancer detection in a biopsy naïve cohort is 99% when taking three cores per target [26]. Moreover, the number of cores taken per target did not affect the rate of GG2-5 PCa detected in Hispanic men. There were no differences in the GG2-5 PCa detection rate in Hispanic men with 2 or fewer cores per target compared to 3 or more cores per target (31.9% vs 33.3%), respectively. Therefore, it is unlikely that this difference in target cores taken would influence and cause the lower sensitivity in Hispanic men compared to White men.

The data presented herein provides further evidence of the additive value of mpMRI in detection of GG2-5 PCa when combined with clinical features, particularly in Black and White men. The sensitivity of PIRADS may vary by race and may be lower for Hispanics. One way to increase the sensitivity of mpMRI in both Hispanic and Black men would be to use PSA density > 0.15 ng/ml^2^ as a biopsy threshold in men with a negative MRI [27]. Although, Hispanic men had a lower PSA density compared to White and Black men, over 80% of all patients in our cohort with a negative MRI and missed GG2-5 PCa had a PSA density > 0.15 ng/ml^2^. Some men with a negative MRI will still get a PB as seen in our cohort. Alternatively, utilizing a PSA density < 0.15 ng/ml^2^ as a cutoff in men with negative MRI to identify men without GG2-5 PCa could have avoided 70% of unnecessary BP in men with negative MRI in our study. Our study further validates the meta-analysis done by Pagniez et al. which states that PSA density < 0.15 ng/ml^2^ is a useful factor to use in men with PIRADS 1–2 lesions to identify men without GG2-5 PCa who could avoid biopsy [27].

One of the strengths of our study is our focus on biopsy-naïve populations and the inclusion of Hispanics. Other studies comparing PIRADS performance and outcomes in diverse populations include patients on Active Surveillance or patients with previous negative biopsies [13, 24, 25]. Our cohort is exclusively biopsy-naïve which lends well to helping urologists make clinical decisions on biopsy-naïve patients that present to clinic.

### Limitations

The limitations of this study include its retrospective nature and small sample size. However, the study involves fellowship trained genitourinary radiologists and pathologists and is the largest data set on Hispanics which is the largest ethnic minority group in the US. Despite including patients who were evaluated mostly based on version 2.0 of the PIRADS system, we acknowledge that like many radiologic modalities, mpMRI readings are subject to interobserver variability [28]. Prospective studies with larger ethnic minority enrollment and with radiology and pathologic consensus or confirmation with radical prostatectomy specimen are encouraged to validate our results.

## Conclusions

The sensitivity of PIRADS may be lower in biopsy-naïve Hispanic men and could potentially delay diagnosis. PIRADS provides orthogonal data to clinical variables in detection of clinically significant prostate cancer in biopsy-naïve patients. Larger prospective validation studies in ethnic minorities are needed.

## Supplementary Information

**Additional file 1:** De-identified patient data with corresponding legend.

## Data Availability

The dataset supporting the conclusions of this article is included within the article (and its additional file).
